# Clinical utility of serum fucosylated fraction of alpha-fetoprotein in the diagnostic of hepatocellular carcinoma: a comprehensive analysis with large sample size

**DOI:** 10.18632/aging.203963

**Published:** 2022-03-20

**Authors:** Aibin Liu, Yanyan Li, Lin Shen, Liangfang Shen, Zhanzhan Li

**Affiliations:** 1 Department of Geriatrics, Xiangya Hospital, Central South University, Changsha, Hunan Province 410008, PR China; 2 Department of Nursing, Xiangya Hospital, Central South University, Changsha, Hunan Province 410008, PR China; 3 Department of Oncology, Xiangya Hospital, Central South University, Changsha, Hunan Province 410008, PR China; 4 National Clinical Research Center for Geriatric Disorders, Xiangya Hospital, Central South University, Changsha, Hunan Province 410008, PR China

**Keywords:** hepatocellular carcinoma, biomarkers, molecular diagnosis, meta-analysis, alpha-fetoprotein

## Abstract

We conducted a comprehensive meta-analysis of the utility of AFP-L3 for the diagnosis of hepatocellular carcinoma, to provide a more accurate estimation for the clinical utility of AFP-L3. We performed online searches in five databases (PubMed, China National Knowledge Infrastructure, Wanfang, Web of Science, and Embase), from inception to December 31, 2021. Pooled sensitivity, specificity, and area under the curve (AUC) with the matching 95% confidence intervals (95% CIs) were calculated to estimate the diagnostic value of AFP-L3. Thirty-four studies were included in the meta-analysis. The pooled sensitivity was 0.70 [95% confidence interval (CI): 0.63–0.77], and the specificity was 0.91 (95% CI: 0.88–0.94). The estimated area under the curve (AUC) was 0.90 (95% CI: 0.87–0.92). The positive likelihood ratio and negative likelihood ratio were 7.78 (95% CI: 5.7–10.7) and 0.33 (95% CI: 0.26–0.41), respectively. The diagnostic odds ratio was 24 (95% CI: 16–37). The subgroup analysis indicated moderate sensitivity (0.79) and high specificity (0.89) for the Asian population (AUC = 0.89), and similar specificity (0.95) but lower sensitivity (0.35) for Caucasians (AUC = 0.80). Deeks’ funnel plot asymmetry test detected no publication bias (*P* = 0.460). The sensitivity analysis showed that the pooled results were stable. Taken together, our results indicated that AFP-L3 demonstrates high diagnostic ability for HCC, especially among Asian populations. AFP-L3 is a useful means for high-volume screening, which can help doctors optimize diagnosis workflow, reduce workload, and improve detection sensitivity. The combination of multiple biomarkers may provide more accurate diagnostic tools for HCC in the future.

## INTRODUCTION

Liver cancer is one of the most common malignancies of the digestive system, ranking 6th in the incidence and 2nd in terms of tumor mortality worldwide, among which hepatocellular carcinoma (HCC) is the most common subtype of primary liver cancer [1]. According to statistics, there are approximately 840,000 new cases and 780,000 deaths worldwide every year. China had approximately 430,000 new cases, accounting for approximately 55% of the global cases and deaths [2]. Therefore, HCC poses a serious threat to human health. Hepatectomy, liver transplantation, and ablative therapy are the possible treatments for liver cancer [3]. However, early stage HCC is easily missed, and most of the cases are in advanced stage at the time of diagnosis. It is estimated that only about 10% of patients have an operation opportunity [4]. Although surgical resection, tumor vascular embolization, and radiofrequency ablation effectively improve the survival rate of patients with HCC, most patients eventually progress to advanced stages of disease due to cancer invasion, and the prognosis is extremely poor [5]. For many advanced HCC patients receiving chemotherapy, chemotherapeutic drug resistance has become a major obstacle to treatment success [6]. Furthermore, the late diagnosis of liver cancer contributes significantly to high mortality. Therefore, early diagnosis and prevention of HCC are crucial for reducing mortality.

Alpha-fetoprotein (AFP) is currently the most widely used diagnostic marker in clinical applications; however, its sensitivity in the diagnosis of HCC is about 60–70%, mainly because the serum AFP levels are not elevated in 20–30% of HCC patients. In addition, several other diseases have been linked to an increase in AFP [7]. For example, patients with hepatitis and liver cirrhosis may have elevated AFP levels. In germ cell tumors, AFP elevation was mainly found in testicular and ovarian embryonal tumors. In pregnant women, AFP levels are increased because this protein is synthesized in the yolk sac [8]. Therefore, AFP is an auxiliary diagnostic marker for HCC, which may result in a certain proportion of omission or misjudgment. Therefore, new tumor biomarkers are needed to assist in the diagnosis of HCC. Taketa et al., found that after binding with *Lens culinaris* agglutinin in the serum of patients with primary hepatocellular carcinoma (HCC), AFP was separated into three bands by electrophoresis, which were named AFP-L1, AFP-L2, and AFP-L3, namely, the unbinding type of LCA (AFP-L1, AFP-L2) and the binding type of LCA (AFP-L3) [9]. AFP-L3 is mainly found in benign liver diseases, such as chronic hepatitis and cirrhosis. It is mostly produced by yolk sac tumors and can also be detected in the serum of pregnant women; AFP-L3 can only be produced by liver tumor cells. Further studies have found that AFP-L3 is a marker of the degree of biological malignancy of HCC [10]. The levels of AFP-L3 in HCC patients were significantly higher than those in patients with other diseases such as cirrhosis [11]. AFP-L3 positivity precedes identification of cancer with B-ultrasound, CT, and other imaging methods, suggesting the existence and occurrence of liver cancer; AFP-L3 is not correlated with AFP level. Therefore, the determination of AFP-L3 levels is crucial for differentiating between benign and malignant liver diseases, and the early diagnosis of liver cancer when there is AFP positivity. Currently, many studies have assessed the diagnostic value of AFP-L3 for HCC; however, these studies presented different results because of sample size and population settings. In the present study, we performed a comprehensive meta-analysis of the importance of AFP-L3 for HCC diagnosis, to provide a more accurate estimation of the clinical utility of AFP-L3.

## MATERIALS AND METHODS

We performed this systematic and meta-analysis in accordance with the Preferred Reporting Items for Systematic Reviews and Meta-Analyses: The PRISMA Statement (Supplementary Material 1).

### Literature search

We performed online searches in the five databases (PubMed, China National Knowledge Infrastructure, Wanfang, Web of Science, and Embase) for the diagnostic ability of the fucosylated fraction of alpha-fetoprotein (AFP-L3) for HCC. The search date was from inception to December 31, 2021. The search language was restricted to English and Chinese. The following search terms were used: fucosylated fraction of alpha-fetoprotein, fucosylated fraction of α-fetoprotein, alpha-fetoprotein, α-fetoprotein, AFP-L3, AFP, alpha-AFP, α-AFP, *Lens culinaris* agglutinin-reactive AFP; The other search terms were hepatocellular carcinoma, HCC, primary hepatic carcinoma, PHC, PHCC, liver cancer, hepatoma, hepatic carcinoma, cancer of liver. The search strategy for each database was provided in the Supplementary Material 2.

### Criteria for inclusion and exclusion

The included studies met the following criteria: (1) included assessment of the diagnostic ability of AFP-L3 for primary HCC; (2) primary HCC diagnosis was performed using a pathological biopsy; (3) AFP-L3 detection in blood samples; (4) provided enough data for further analysis. The remaining studies were excluded based on following criteria: (1) irrelevant study topic; (2) no enough data for meta-analysis; (3) the diagnosed tumor was not primary; (4) reviews, letters, comments, duplicates records, and case reports; (5) small sample sizes (less than 30 in statistics). According to these inclusion and exclusion criteria, two scientists independently screened the search results. We first removed duplicated records, excluded irrelevant study topics via scanning titles and abstracts, and further read the full texts for eligibility records.

### Data collection

Using a standard EXCEL sheet, two researchers independently extracted data from the included studies. Disagreements were resolved by consensus. For each study, the following data were extracted: surname of the first author, publication year, gold standard for diagnosing HCC, method for detecting AFP-L3 (ELISA or ECLIA), tumor type, the number of cases and normal controls, mean age or age range of case and control population, four-fold data including true positive (TP), false positive (FP), false negative (FN), and true negative (TN), and the sensitivity and specificity of each study.

### Assessment of quality

The Quality Assessment of Diagnostic Accuracy Studies-2 is a tool recommended by the Cochrane Library Handbook for assessing diagnostic quality [12]. This tool addresses the risk of bias and applicability concerns. The risk of bias included patient selection, index test, reference standard, flow and timing, and applicability concerns including patient selection, index test, and reference standard. Risk of bias was classified as low, unclear, and high risk of bias, and items of applicability concerned were classified as low, unclear, and high concern. If one of the sub-items was high risk or concerned, the whole item was judged as high risk or concerned.

### Statistical analysis

We first assessed the threshold effect of data that determined whether we will use a bivariate analysis [13]. The heterogeneity within studies was assessed using the Chi-square test and I^2^ statistics. *P* < 0.05, and I^2^ > 50% indicated significant heterogeneity. The random-effect model was used for significant heterogeneity, and the fixed-effect model was used for no heterogeneity [14, 15]. The diagnostic ability of AFP-L3 was assessed using the pooled sensitivity, specificity, positive likelihood ratio (PLR), negative likelihood ratio (NLR), estimated area under the curve (AUC), and diagnostic odds ratio (DOR) [16]. For AUC, a value of 1.0 was considered as the highest diagnostic accuracy, and an AUC < 0.5 indicated a poor diagnostic accuracy [17–19]. Subgroup analysis was performed for different publication years (median of publication year: before 2012 vs. after 2011), sample sizes (median: >150 vs. <151), and study populations (Asian vs. Caucasian). Fagan’s plot was used to assess the relationship between pre-test probability and post-test probability. Publication bias was assessed using the Deeks’ funnel plot asymmetry test [20]. Sensitivity analysis was assessed using four methods: goodness of fit, bivariate normality, influence analysis, and outlier detection. All analyses were completed using Stata software version 14.0 and Rev Manager 5.3. *P* < 0.05 was considered significant unless otherwise specified.

### Data availability

All data was within the manuscript without any restriction.

### Ethnics approval and consent to participate

The ethics approval and consent to participate are not applicable because this is a meta-analysis.

## RESULTS

### Search results and study selection

**Figure 1:**
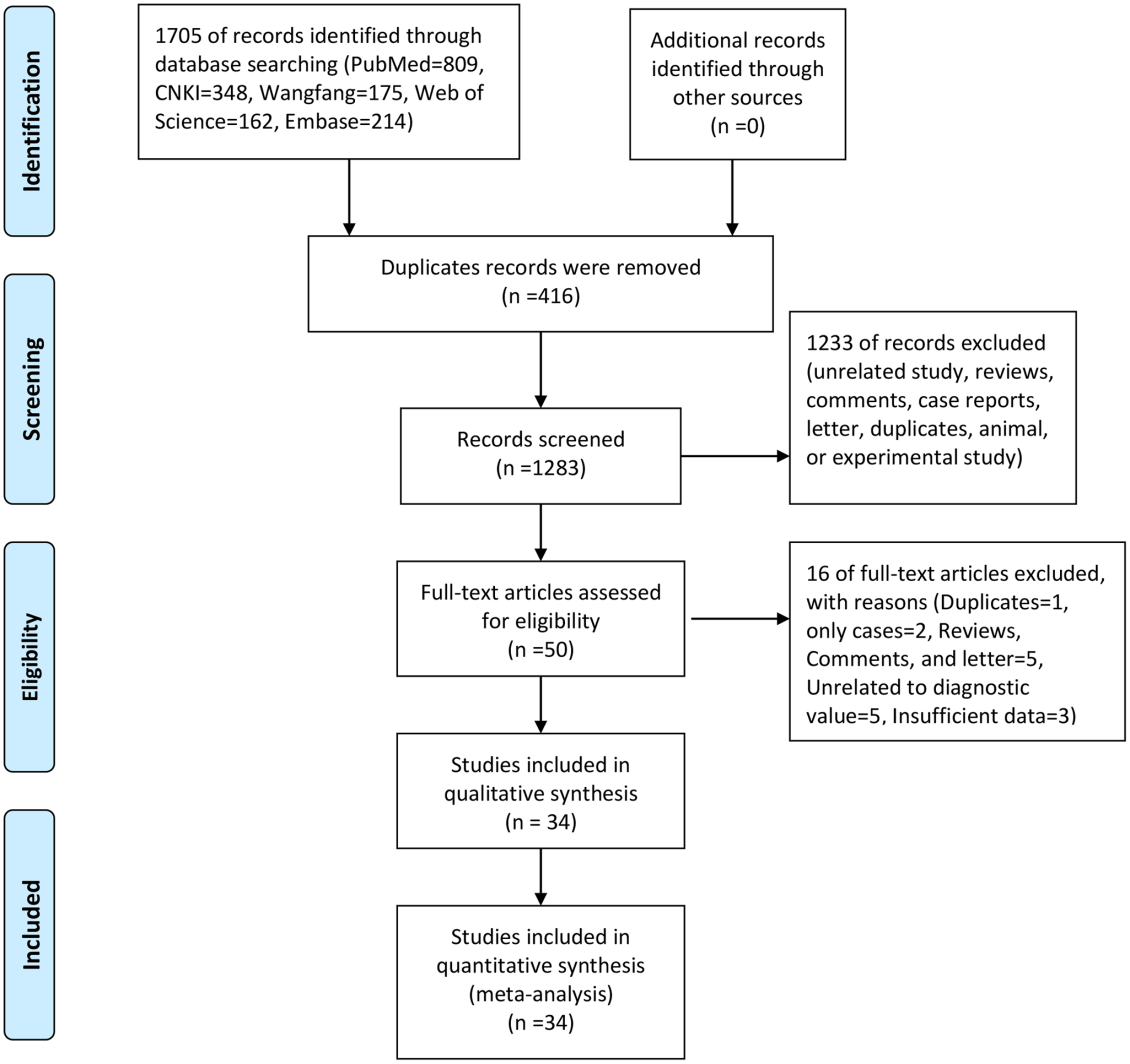
The flow chart of study selection.

The initial search obtained 1,708 articles from five databases (PubMed = 809, CNKI = 348, Wanfang = 175, Web of Science = 162, Embase = 214). No articles were identified from other resources. After removing 416 duplicates, 1,293 articles were analyzed. We then read the titles and abstracts for further screening, and 1,233 records, including obviously unrelated studies, reviews, comments, case reports, letters, animal and experimental studies, were excluded. Fifty full-text articles were used for the eligibility assessment. One duplicate, two case reports, five reviews, comments, and letters, and eight studies with unrelated or insufficient data were excluded. Finally, 34 studies were included in the qualitative and quantitative synthesis analyses (Supplementary Material 3). Figure 1 presents the study selection process.

### Study characteristics

Thirty-four studies were published from 2002 to 2020. All studies used pathology results as the gold standard for diagnosis, and all patients had primary tumors. Three studies used the enzyme-linked immunosorbent assay (ELISA) for analyzing AFP-L3, and the rest of them used the electrochemical luminescent immunoassay (ECLIA). All studies considered 10% as the cutoff value for diagnosing HCC. The sample size ranged from 80 to 666. The total population was 7,422, including 3,498 cases and 3,924 controls. The sensitivity of a single study ranged from 0.13 to 0.91, and the specificity ranged from 0.62 to 1.00. Table 1 presents the details of the extracted data.

**Table 1:** General characteristics of included studies.

**Author**	**Year**	**Gold standard**	**Cut-off value**	**Method for examination**	**Tumor Type**	**Case**		**Control**		**TP**	**FP**	**FN**	**TN**	**Sensitivity**	**Specificity**
						***n***	**age**	***n***	**age**						
Zhou	2020	Pathology	10	ELISA	Primary	300	58.3	100	58.4	214	25	86	75	0.71	0.75
Jiao	2019	Pathology	10	ECLIA	Primary	52	53.9	58	52.3	31	22	21	36	0.60	0.62
Wang	2019	Pathology	10	ELISA	Primary	82	56.9	241	57.6	51	16	31	241	0.62	0.93
Xu	2007	Pathology	10	ECLIA	Primary	33	16–67	54	–	30	0	3	59	0.91	1.00
Zheng	2009	Pathology	10	ECLIA	Primary	45	52.0	84	50.0	41	6	4	78	0.91	0.93
Han	2012	Pathology	10	ECLIA	Primary	92	–	45	–	79	2	13	43	0.86	0.96
Sun	2008	Pathology	10	ELISA	Primary	79	19–67	53	19–67	67	4	12	49	0.85	0.92
Ma	2011	Pathology	10	ECLIA	Primary	75	11–82	95	11–82	62	22	13	73	0.83	0.77
Han	2018	Pathology	10	ECLIA	Primary	85	15.2	38	43.1	70	5	15	33	0.82	0.87
Lu	2014	Pathology	10	ECLIA	Primary	90	47.1	60	48.1	69	10	21	50	0.77	0.83
Chen	2012	Pathology	10	ECLIA	Primary	176	8–83	251	10–80	148	63	28	188	0.84	0.75
Zhou	2015	Pathology	10	ECLIA	Primary	170	47.5	130	48.0	143	26	27	104	0.84	0.80
Ma	2011	Pathology	10	ECLIA	Primary	75	11–82	95	11–82	62	22	13	73	0.83	0.77
Li	2013	Pathology	10	ECLIA	Primary	185	41.8	225	33.4	155	17	30	208	0.84	0.92
Niu	2010	Pathology	10	ECLIA	Primary	69	–	82	–	56	7	14	75	0.80	0.91
Wang	2007	Pathology	10	ECLIA	Primary	47	47.3	83	47.3	34	2	13	81	0.72	0.98
Li	2017	Pathology	10	ECLIA	Primary	40	47.6	40	58.2	22	2	18	38	0.55	0.95
Zhang	2019	Pathology	10	ECLIA	Primary	30	50.4	60	53.0	18	15	12	45	0.60	0.75
Jia	2010	Pathology	10	ECLIA	Primary	88	52.0	72	52.0	66	6	22	66	0.75	0.92
Wang	2020	Pathology	10	ECLIA	Primary	83	51.4	179	50.1	63	52	20	127	0.76	0.71
Zhu	2020	Pathology	10	ECLIA	Primary	40	67.6	40	68.5	34	9	6	31	0.85	0.77
Cao	2013	Pathology	10	ECLIA	Primary	43	–	40	–	35	3	8	37	0.81	0.93
Cheng	2017	Pathology	10	ECLIA	Primary	79	47.0	186	45.3	65	14	14	172	0.82	0.92
Huang	2012	Pathology	10	ECLIA	Primary	92	22–87	45	22–87	77	5	13	40	0.86	0.89
Zeng	2020	Pathology	10	ECLIA	Primary	50	45.6	100	46.2	38	7	12	93	0.76	0.93
Jiang	2009	Pathology	10	ECLIA	Primary	56	–	60	–	36	0	20	60	0.64	0.98
Tamura	2010	Pathology	10	ECLIA	Primary	295	70	350	60	113	2	182	348	0.38	0.99
Nouso	2011	Pathology	10	ECLIA	Primary	196	70.2	87	71.4	26	10	170	77	0.13	0.89
Toyoda	2011	Pathology	10	ECLIA	Primary	270	67.9	396	63.5	40	7	230	389	0.15	0.98
Durazo	2008	Pathology	10	ECLIA	Primary	144	58.3	96	58.3	81	10	63	86	0.58	0.90
Leerapun	2007	Pathology	10	ECLIA	Primary	166	60.5	106	–	80	13	86	93	0.48	0.88
Zinkin	2008	Pathology	10	ECLIA	Primary	41	60	51	60	26	3	15	48	0.63	0.94
Sterling	2009	Pathology	10	ECLIA	Primary	74	54.9	298	54.9	27	25	47	273	0.36	0.92
Shimizu	2002	Pathology	10	ECLIA	Primary	56	62	34	60	22	1	34	33	0.39	0.97

Abbreviations: ELISA: enzyme linked immunosorbent assay; ECLIA: electrochemiluminescence; TP: True positive, TN: True negative, FP: False positive, FN: False negative, FP: False positive, TN: True negative.

### Risk of bias within studies

**Figure 2:**
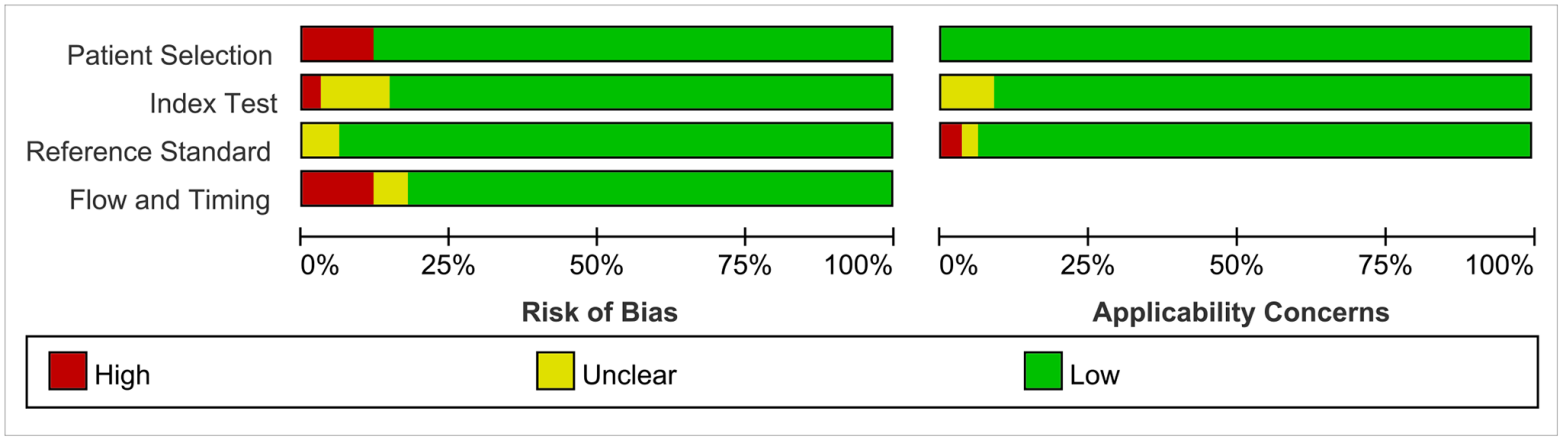
Risk of bias and applicability concerns graph.

**Figure 3:**
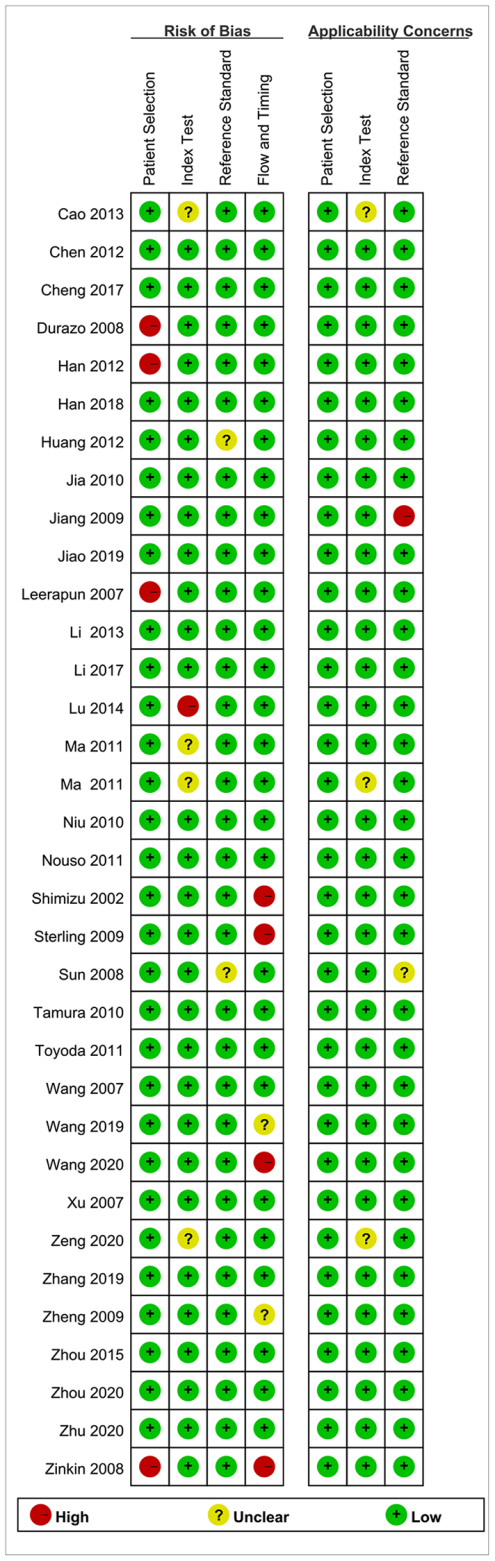
Risk of bias and applicability concerns summary.

Figures 2 and 3 present the methodological quality graph and the methodological quality summary. As we can see, three studies were considered having a high risk of bias due to the patient’s selection, index text, flow, and timing. One study was considered having a high applicability concern because of the reference standard. The single item rate with risk of bias was less than 10%. Four, two, and two studies had unclear risk of bias at the level of index test, the reference standard and flow and timing, respectively. Three studies at the level of index test and one study at the level of reference standard have unclear applicability concerns. The rate of unclear risk of bias was 23.5%. Overall, the quality of the included studies was relatively high.

### Synthesis of results

**Figure 4:**
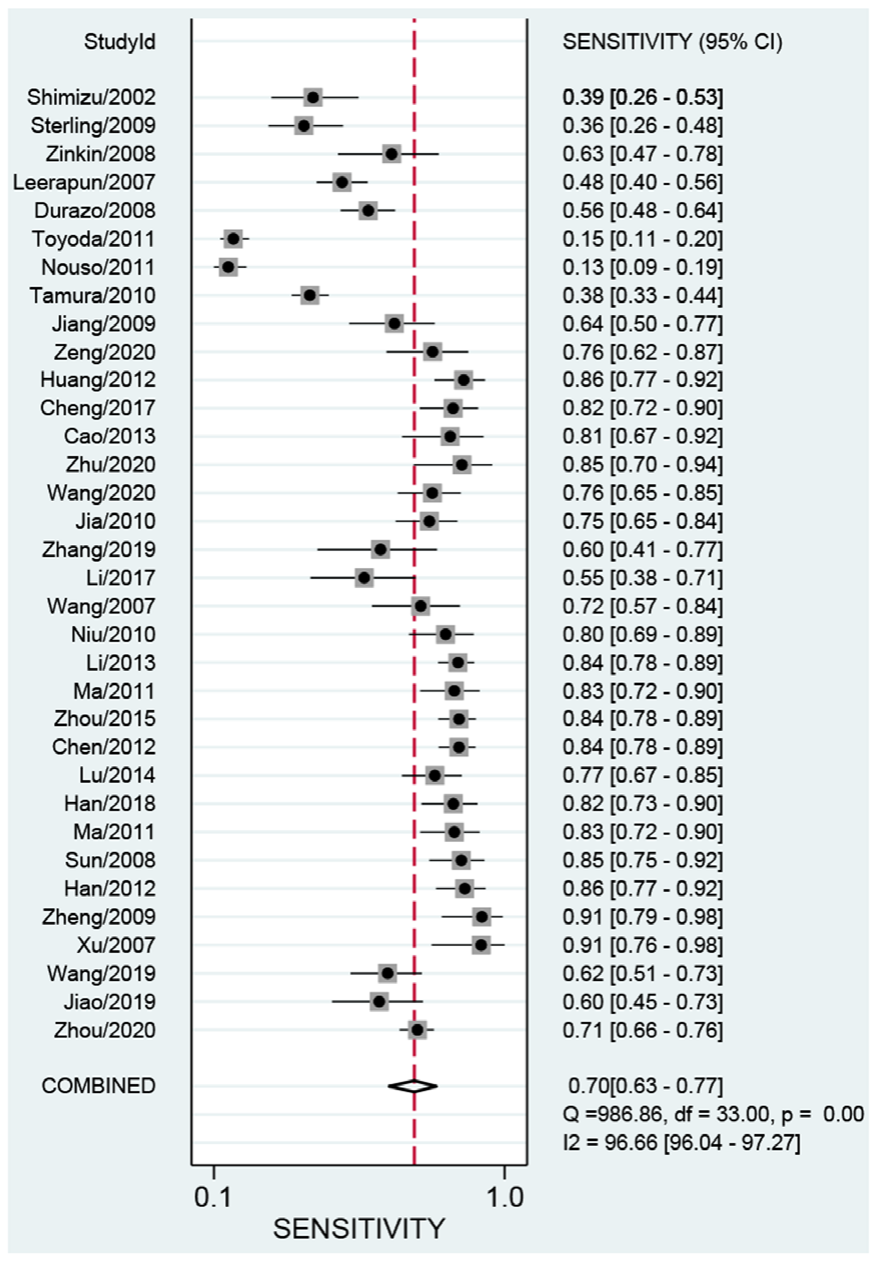
Pooled sensitivity of AFP-L3 in diagnosing HCC.

**Figure 5:**
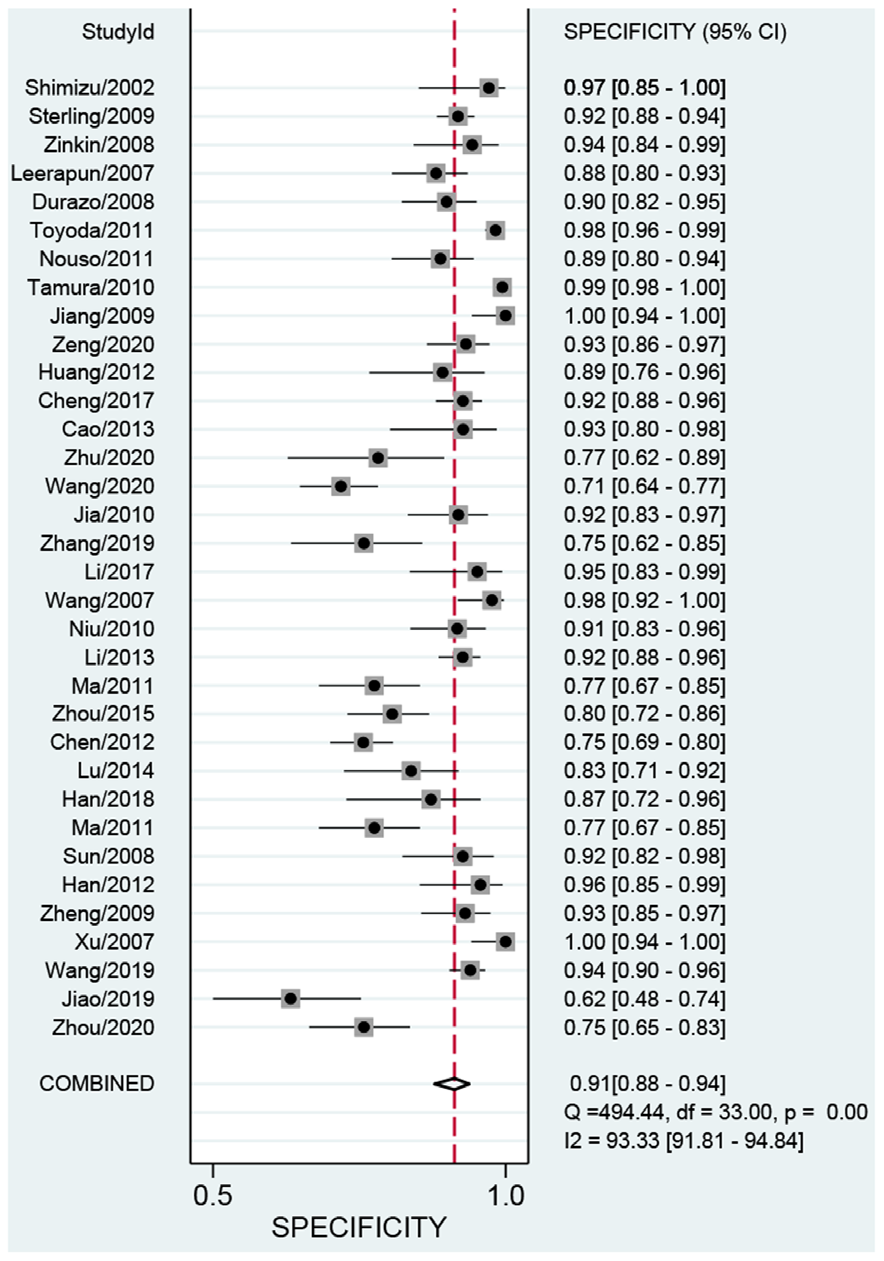
Pooled specificity of AFP-L3 in diagnosing HCC.

**Figure 6:**
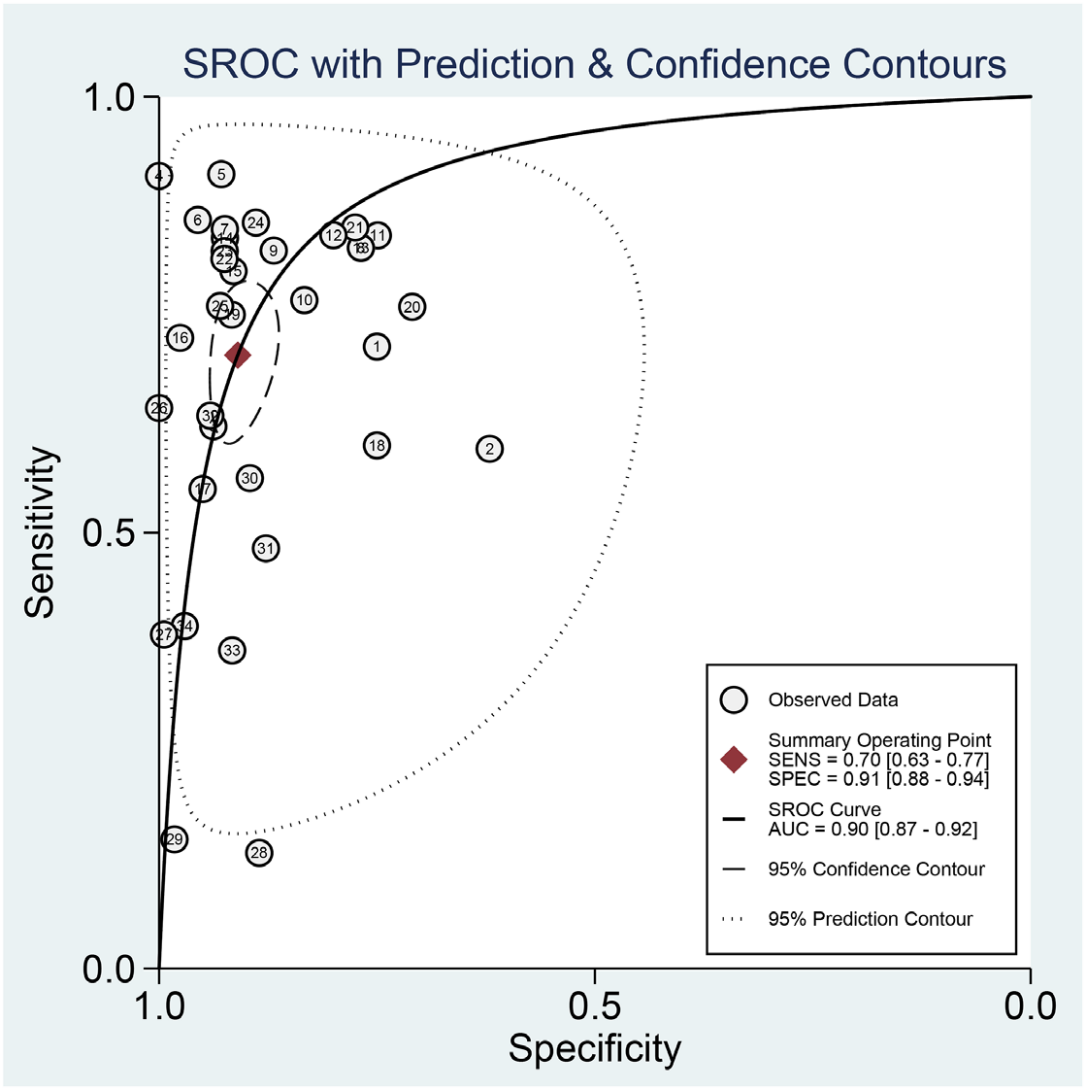
The SROC curve of AFP-L3 for HCC.

**Figure 7:**
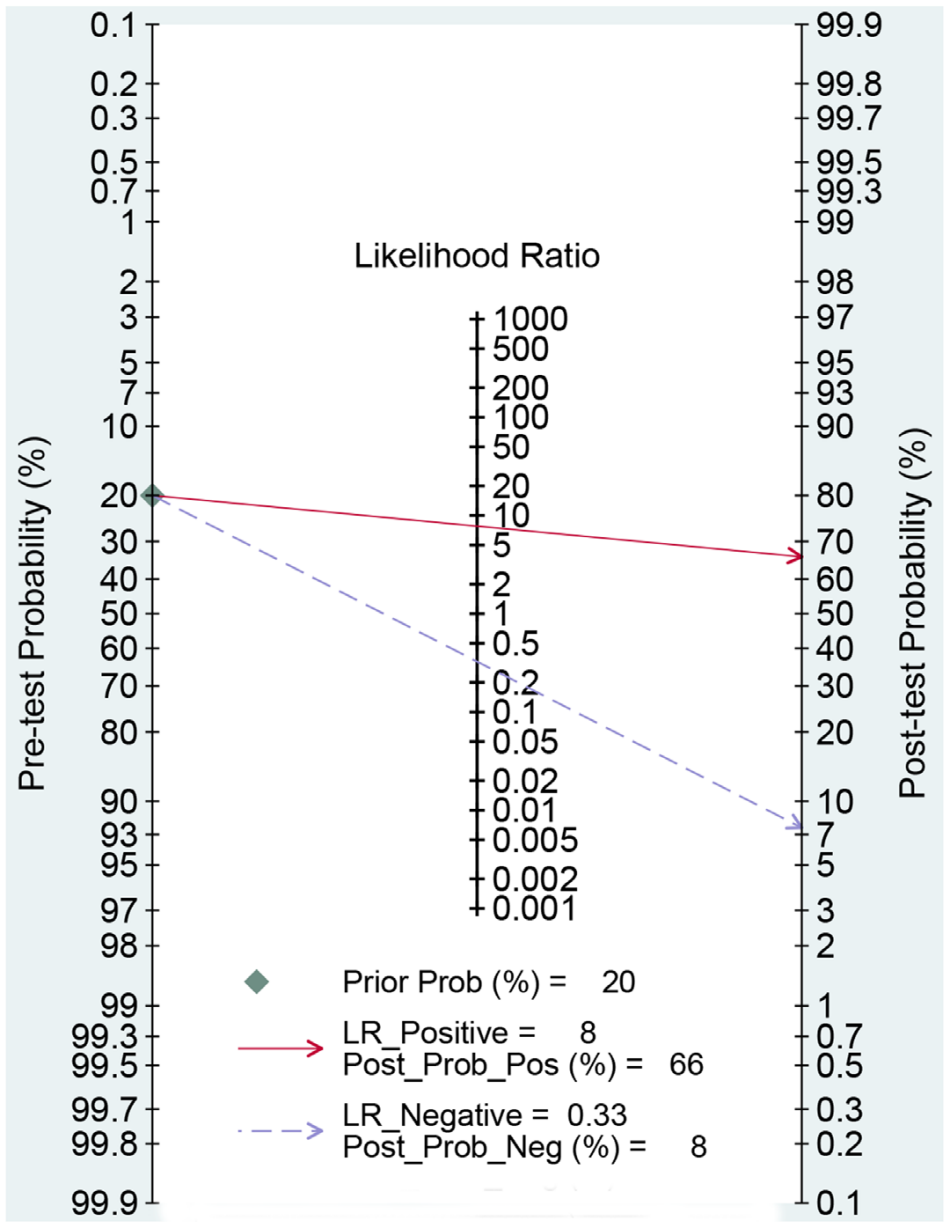
Fagan diagram assessing the overall diagnostic value of AFP-L3 for HCC.

**Figure 8:**
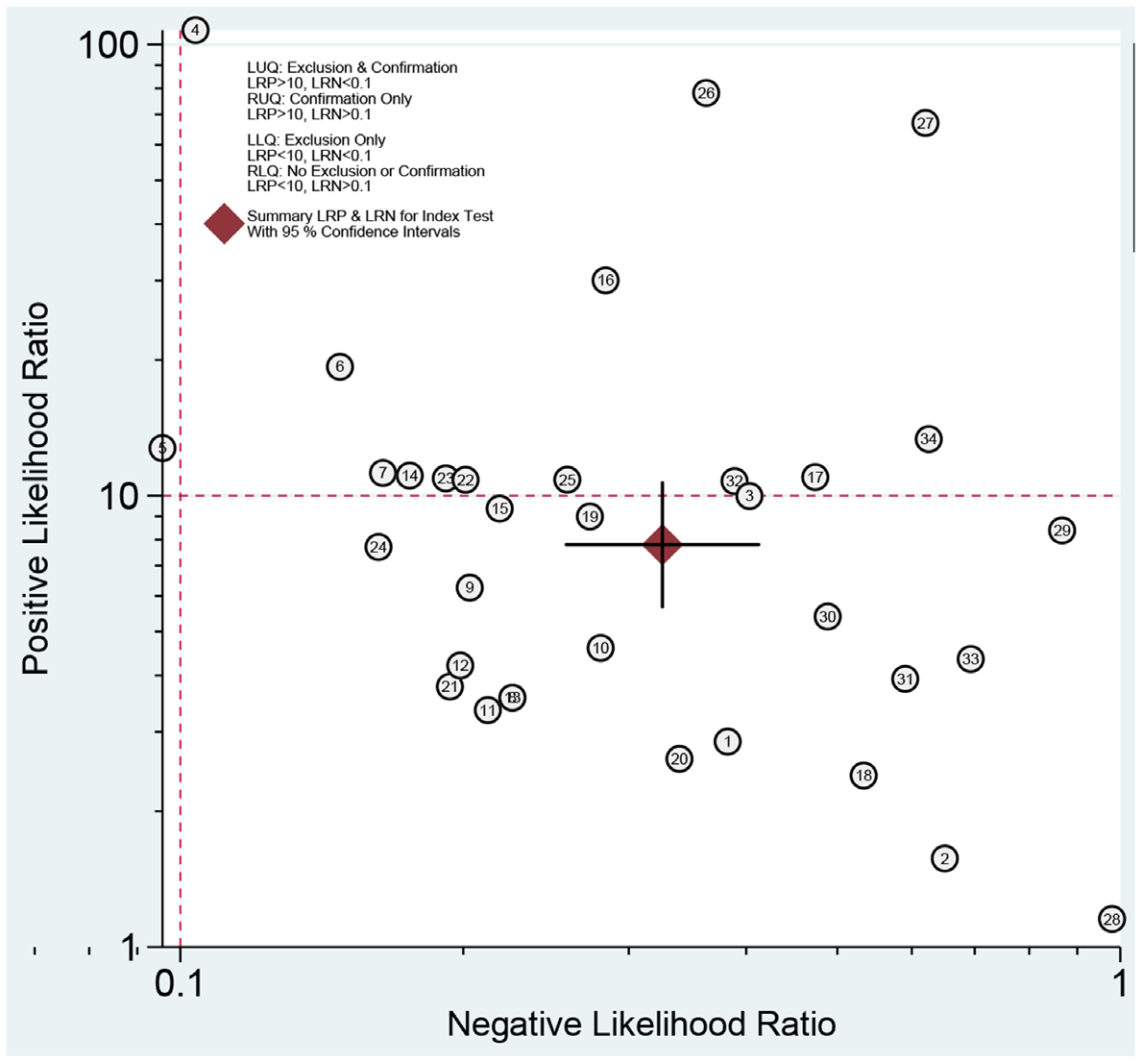
Summary LRP and LRN for index test with 95% CI.

We first performed a threshold effect test, and the Spearman coefficient indicated there was no threshold effect (r = 0.212, *P* = 0.244). The pooled sensitivity was 0.70 (95% CI: 0.63–0.77, Figure 4), and the specificity was 0.91 (95% CI: 0.88–0.94, Figure 5). The estimated AUC was 0.90 (95% CI: 0.87–0.92, Figure 6). The PLR and NLR were 7.78 (95% CI: 5.7–10.7) and 0.33 (95% CI: 0.26–0.41), respectively. The DOR was 24 (95% CI: 16–37). Figure 7 presents the Fagan plot. If the pre-test probability of one person was 20% for HCC diagnosis, then the post-test probability would be 66%, with a likelihood ratio of 8. If the pre-test probability of one individual was 20%, the post-test probability would be 8%, with a likelihood ratio of 0.33 (Figure 7). Figure 8 presents the scatter matrix. We can see that the scatter was mainly distributed in LRP < 10, and the LRN < 0.1 consisted of few scatters, which means that AFP-L3 tends to be used for exclusion.

We also performed subgroup analyses for publication year, sample size, and study population (Table 2). Before 2012, the pooled sensitivity, specificity, and AUC were 0.62 (95% CI: 0.48–0.75), 0.94 (95% CI: 0.91–0.97), and 0.92 (95% CI: 0.89–0.94), respectively. The PLR and NLR were 11.3 (95% CI: 6.6–19.2) and 0.40 (95% CI: 0.28–0.57), respectively. The diagnostic OR was 28 (95% CI: 14–59). For studies after 2011, the pooled sensitivity, specificity, and AUC were 0.78 (95% CI: 0.73–0.82), 0.86 (95% CI: 0.81–0.90), and 0.88 (95% CI: 0.85–0.90), respectively. The PLR and NLR were 5.6 (95% CI: 4.0–7.8) and 0.26 (95% CI: 0.21–0.32), respectively. The diagnostic OR was 21 (95% CI: 13–34). For studies with sample size >150, the pooled sensitivity, specificity, and AUC were 0.65 (95% CI: 0.51–0.76), 0.90 (95% CI: 0.84–0.93), and 0.88 (95% CI: 0.85–0.90), respectively. The PLR and NLR were 6.2 (95% CI: 4.4–8.9) and 0.26 (95% CI: 0.21–0.32), respectively. The DOR was 21 (95% CI, 13–34). For studies with sample size <151, the pooled sensitivity, specificity, and AUC were 0.76 (95% CI: 0.69–0.82), 0.93 (95% CI: 0.88–0.96), and 0.91 (95% CI: 0.88–0.93), respectively. The PLR and NLR were 10.2 (95% CI: 6.0–17.3) and 0.26 (95% CI: 0.20–0.34), respectively. The DOR was 39 (95% CI, 20–77). For the Asian population, the pooled sensitivity and specificity were 0.79 (95% CI: 0.75–0.82) and 0.89 (95% CI: 0.85–0.92), respectively. The AUC was 0.89 (95% CI: 0.86–0.91). The PLR and NLR were 7.0 (95% CI: 5.1–9.6) and 0.24 (95% CI: 0.20–0.28), respectively. The DOR was 29 (95% CI: 19–44). For Caucasians, the data of three studies showed that the pooled sensitivity and specificity were 0.36 (95% CI: 0.25–0.50) and 0.95 (95% CI: 0.90–0.98). The AUC was 0.80 (95% CI: 0.76–0.83). The PLR and NLR were 7.4 (95% CI: 3.6–15.4) and 0.67 (95% CI: 0.55–0.82). The DOR was 11 (95% CI: 5–26).

**Table 2:** Summary estimated of diagnostic performance of AFP-L3 for HCC.

**Category**	**Sensitivity (95% CI)**	**Specificity (95% CI)**	**AUC (95% CI)**	**PLR (95% CI)**	**NLR (95% CI)**	**DOR (95% CI)**
Overall	0.70 (0.63–0.77)	0.91 (0.88–0.94)	0.90 (0.87–0.92)	7.8 (5.7–10.7)	0.33 (0.26–0.41)	24 (16–37)
**Publication year**						
Before 2012	0.62 (0.48–0.75)	0.94 (0.91–0.97)	0.92 (0.89–0.94)	11.3 (6.6–19.2)	0.40 (0.28–0.57)	28 (14–59)
After 2011	0.78 (0.73–0.82)	0.86 (0.81–0.90)	0.88 (0.85–0.90)	5.6 (4.0–7.8)	0.26 (0.21–0.32)	21 (13–34)
**Sample size**						
>150	0.65 (0.51–0.76)	0.90 (0.84–0.93)	0.88 (0.85–0.90)	6.2 (4.4–8.9)	0.40 (0.28–0.55)	16 (10–25)
<151	0.76 (0.69–0.82)	0.93 (0.88–0.96)	0.91 (0.88–0.93)	10.2 (6.0–17.3)	0.26 (0.20–0.34)	39 (20–77)
**Study population**						
Asian	0.79 (0.75–0.82)	0.89 (0.85–0.92)	0.89 (0.86–0.91)	7.0 (5.1–9.6)	0.24 (0.20–0.28)	29 (19–44)
Caucasian	0.36 (0.25–0.50)	0.95 (0.90–0.98)	0.80 (0.76–0.83)	7.4 (3.6–15.4)	0.67 (0.55–0.82)	11 (5–26)
Influence analysis	0.70 (0.62–0.78)	0.92 (0.89–0.94)	0.91 (0.88–0.93)	8.6 (6.3–11.8)	0.32 (0.25–0.41)	27 (17–41)
Outlier detection	0.70 (0.62–0.78)	0.91 (0.88–0.94)	0.91 (0.88–0.93)	8.2 (6.1–11.2)	0.32 (0.25–0.42)	25 (17–38)

Abbreviations: AUC: area under the curve; PLR: positive likelihood ratio; NLR: negative likelihood ratio; DOR: diagnostic odds ratio; HCC: hepatocellular carcinoma.

### Publication bias

**Figure 9:**
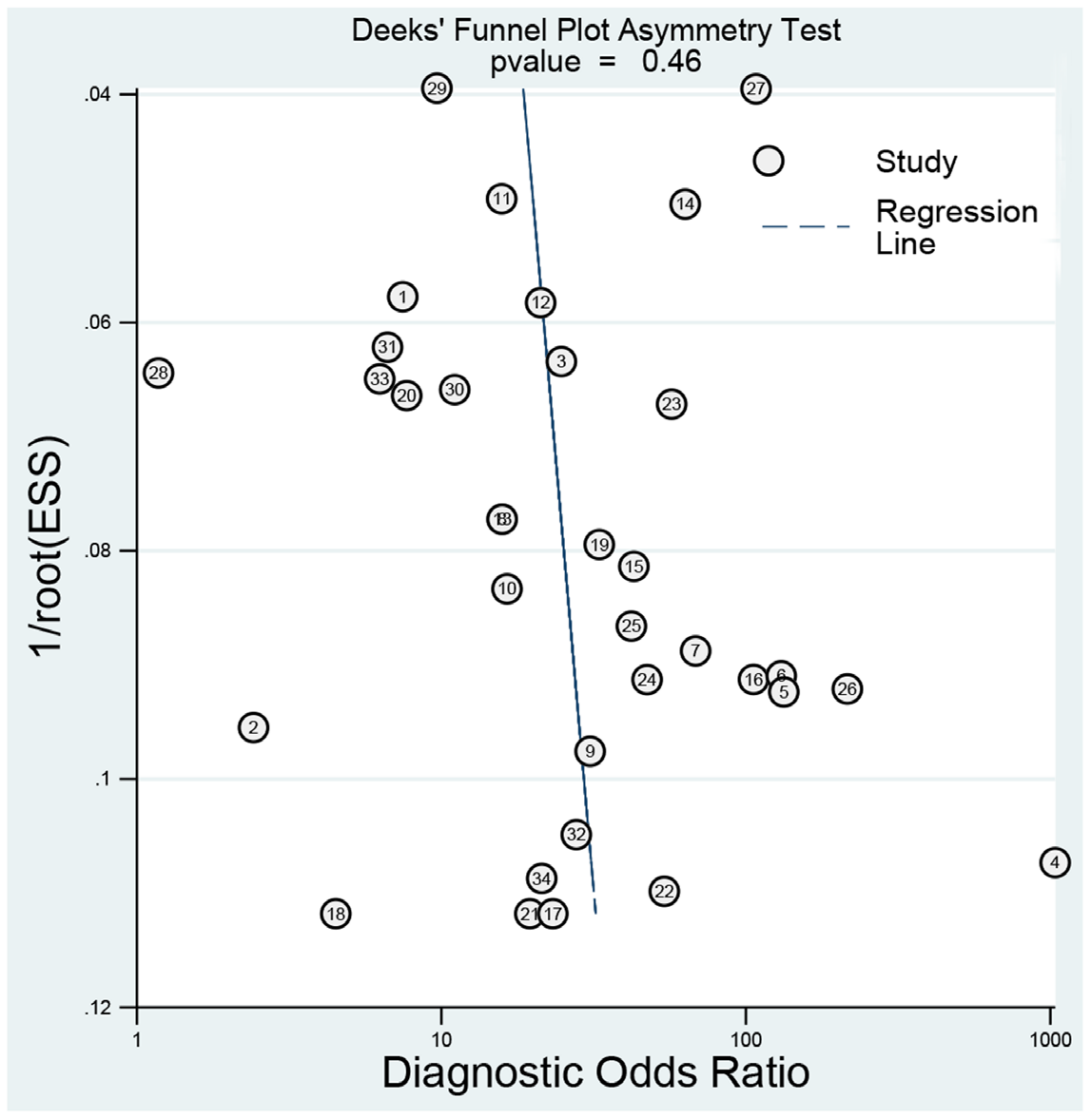
Deeks’ plot for publication bias.

We used the Deeks’ funnel plot asymmetry test to detect publication bias. As shown in Figure 9, the regression line is almost vertical, and the test results indicate no publication bias (*P* = 0.460).

### Sensitivity analysis

**Figure 10:**
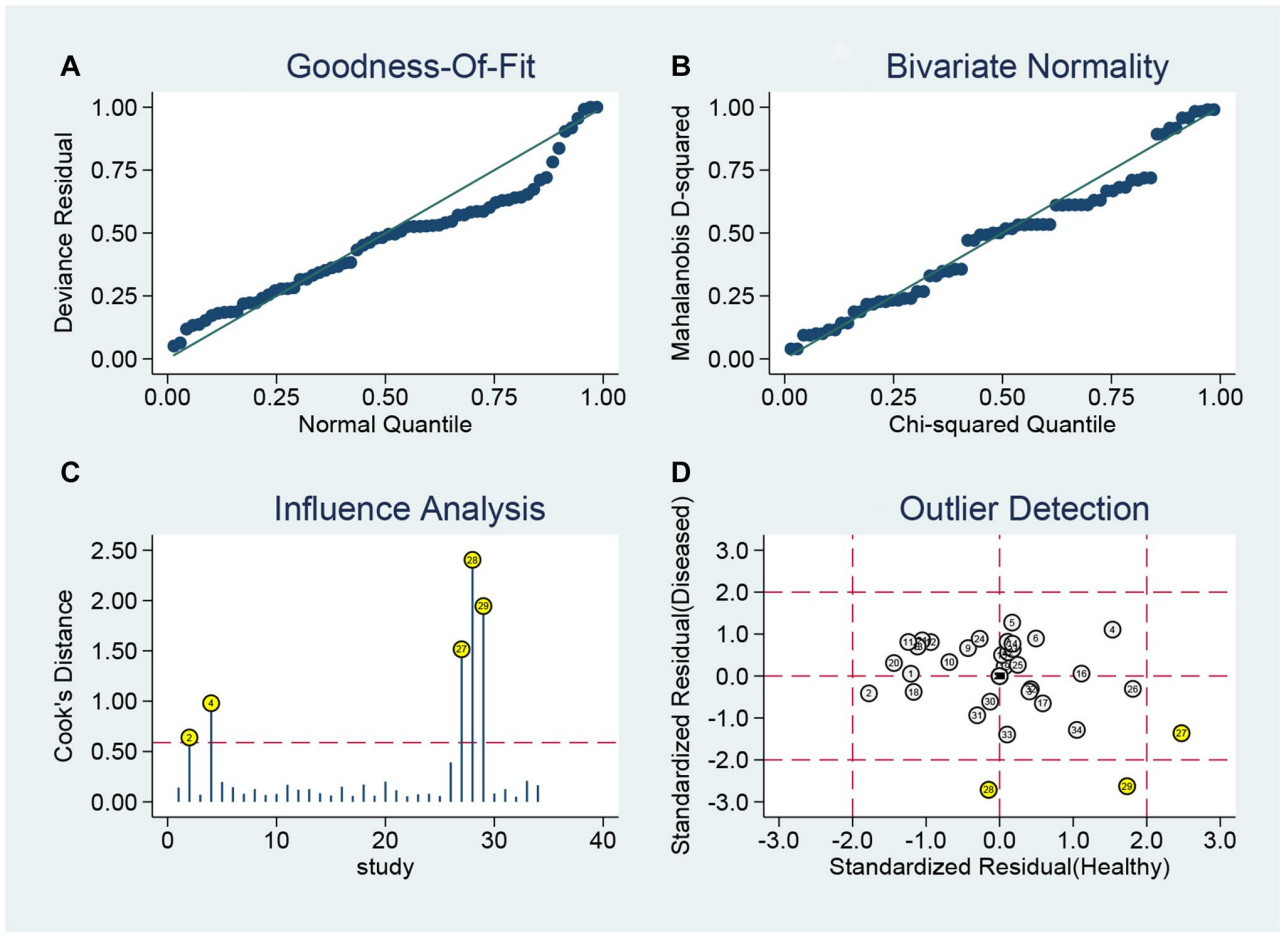
Sensitivity analyses: graphical depiction of residual based goodness-of-fit (**A**), bivariate normality (**B**), and influence analysis (**C**), and (**D**) outlier detection analysis.

The sensitivity analysis is presented in Figure 10. The goodness of fit (Figure 10A) and bivariate normality (Figure 10B) show the degree of fitting of the regression line to the observed value. As shown, the observed value is distributed around the reference line. The observed values are stable. The influence analysis (Figure 10C) indicated that four studies may affect the pooled results. The outlier detection (Figure 10D) indicated that three studies were out of the detection range. After excluding these studies, the pooled sensitivity, specificity, and AUC were 0.70, 0.92, and 0.91, respectively (Table 2). The outlier detection test indicated that the pooled results was very close to the overall results. Overall, the pooled results were still stable.

## DISCUSSION

The present study indicates that the diagnostic ability of AFP-L3 for HCC is high. The AUC was 0.91. Specifically, the sensitivity was moderate (0.70) and the specificity was high (0.90). High sensitivity and specificity were found among Asians, and similar specificity but lower sensitivity was found among Caucasians. AFP-L3 could efficiently be used for excluding non-HCC persons. The subgroup analyses indicated that the diagnostic ability was heterogeneous and affected by publication year. The sample size did not affect the pooled results. The multiple sensitivity analyses suggested that the present results are stable.

The clinical application of AFP-L3 began in 2002 [21]. Initially, some studies explored the cutoff value of AFP-L3, and different diagnostic cutoff values were used. Then, the cutoff value of AFP-L3 was defined as 10% for HCC diagnosis [22]. A previous meta-analysis assessed the diagnostic ability of AFP-L3 for HCC in 2013 [22]. However, there are several differences between the present study and the previous study that need to be addressed. First, our study included more studies and larger sample sizes. The previous study only included 12 studies with a sample size of 2,245, whereas our current study comprised 39 studies with a sample size of 7,422. Second, the previous meta-analysis included studies with different diagnostic cutoff values of AFP-L3 ranging from 3% to 28.3%, whereas we used the same cutoff value of AFP-L3 (10%), which means that our pooled results are universal. Third, the previous study did not avoid the case-control design of a diagnostic test, which introduces a risk of bias in study assessment, while the present meta-analysis included only four case-control studies. Finally, we performed subgroup analysis in different populations, sample sizes, and publication years; additionally, sensitivity analyses were also performed. Therefore, our study provides additional information.

AFP has been used as a serologic diagnostic biomarker for primary HCC, as it was found to be elevated in patients with liver cancer in the 1970s [23]. However, there are some limitations in the application of this biomarker in early HCC screening. For example, AFP levels are not significantly increased in about 30–40% of confirmed liver cancer patients, while AFP is increased in some non-liver cancer patients [24]. Due to the low sensitivity and specificity of AFP in the screening and diagnosis of HCC, the European Association for the Study of The Liver and the American Association for the Study of Liver Diseases no longer suggest AFP as a screening and diagnosis standard in their updated guidelines for the diagnosis and treatment of liver cancer [25]. Previous studies have attempted to improve the sensitivity and specificity using the combination of several biomarkers to achieve early diagnosis and treatment, and to improve the survival prognosis of patients [26]. However, the ideal tumor markers should not only have high specificity, but also high sensitivity, which can allow diagnosis of liver cancer in the early stage. In addition, they should be easy to detect, repeatable, and less invasive. At the same time, they also need to have high sensitivity, which can allow diagnosis in the early stage of liver cancer, and the characteristics of easy detection, repeatability, and less invasion. AFP-L3 has these advantages. It is derived from cancerous hepatocytes and has high specificity for HCC. It may be elevated in the blood of patients with hepatocellular carcinoma, months or even years before imaging studies can reveal the characteristic space-occupying lesions of HCC. It was found that the increase in AFP-L3 occurred 3–28 months earlier than a positive indication from an imaging examination, and the accuracy rate of predicting the occurrence of HCC was 94% [27]. AFP-L3 detection can be used as an early warning of the occurrence of liver cancer in low concentration positive cases of AFP when imaging examination has not found the characteristic space occupying lesions of HCC.

The levels of AFP-L3 are related to malignant characteristics of HCC, especially portal invasion and tumor differentiation. In addition, AFP-L3 levels are associated with the prognosis of liver cancer [28]. After radical resection of HCC, AFP generally turns negative within 2 months, and AFP-L3 also disappears. If AFP is not decreased significantly and does not become negative after a period, and the change in AFP-L3 is not obvious, the operation may be incomplete; there may be residual tumor at the excision edge, vascular cancer plug, satellite nodules, or extrahepatic metastasis. This may explain why AFP-L3 can be used as an early diagnostic marker of HCC.

Other diagnostic markers, such as Golgi protein 73 (GP73), have also been applied in clinical practice. GP73 has been found to be superior to AFP in the diagnosis of early HCC [29]. A study has found that the sensitivity and specificity of GP-73 in diagnosing HCC were 65% and 90%, respectively, which was slightly lower than that of APF-L3 [30]. Alpha-l-fucosidase (AFU) is also a diagnostic marker closely related to HCC, but AFU needs to be combined with AFP to achieve better diagnostic results [31]. Des-gamma-carboxy prothrombin (DCP) is also a serum tumor marker for HCC. Studies on patients with liver cirrhosis and liver cancer have shown that the sensitivity and specificity of DCP in the diagnosis of HCC were 48–62% and 82–98%, respectively [32]. There was no significant correlation between DCP and AFP levels. However, it has been reported that DCP may be associated with intrahepatic metastasis of HCC [33]. Therefore, DCP has no advantage in predicting the prognosis of liver cancer due to AFP, but is advantageous for early diagnosis. In addition, CA19-9 has also been reported as a marker of HCC, but it also needs to be used in combination with AFP to improve diagnosis [34]. The study also found that the results obtained by combined detection of AFP, AFP-L3, and GP73 were much better than those with a single detection. Therefore, GP73 and AFP-L3 are new generation markers for liver cancer diagnosis that can be used in combination to provide better clinical guidance [35]. Our study found that AFP-L3 has high sensitivity and specificity in Asians, but high specificity and lower sensitivity in Caucasians. The reason may be that liver cancer in Europe and the United States is mainly caused by HCV infection, alcohol, and metabolic factors, while HCC in China is mostly associated with chronic HBV infection. Although AFP-L3 has higher diagnostic efficacy than AFP in liver cancer, AFP still has a good diagnostic value in some specific types of liver cancer, such as HBV-associated liver cancer [36].

Some study limitations should be addressed. First, the heterogeneity within studies is high, and the subgroup analyses seem not to decrease the high heterogeneity. Studies based on multicenter studies could be more accurate. Second, most of studies were from Asian population, and the specific population setting is required for accurate estimation. Third, some studies were high-risk bias, which may affect the pooled results. Finally, the combined with other markers could improve the diagnostic efficacy.

## CONCLUSIONS

In conclusion, our results indicated that AFP-L3 has high diagnostic efficacy for HCC, especially among Asian populations. AFP-L3 is useful for high-volume screening, which helps doctors optimize the diagnosis workflow, reduce workload, and improve detection sensitivity. The combination of multiple biomarkers may provide more accurate diagnostic tools for HCC in the future.

## Supplementary Materials

Supplementary Materials 1-3
