# Supplementary material for: Clinical utility of serum fucosylated fraction of alpha-fetoprotein in the diagnostic of hepatocellular carcinoma: a comprehensive analysis with large sample size
Source: Aging (Albany NY). 2022 Mar 20;14(6):2645–64. doi: 10.18632/aging.203963 (PMC9004564; doi:10.18632/aging.203963)
Supplement: Supplementary Materials 1-3 [file aging-14-203963-s001.pdf]

SUPPLEMENTARY MATERIALS

Supplementary Material 1. PRISMA Checklists.

Section/topic	#	Checklist item	Reported on page #
**TITLE**			
Title	1	Identify the report as a systematic review, meta-analysis, or both.	Title (P1)
**ABSTRACT**			
Structured summary	2	Provide a structured summary including, as applicable: background; objectives; data sources; study eligibility criteria, participants, and interventions; study appraisal and synthesis methods; results; limitations; conclusions and implications of key findings; systematic review registration number.	Not applicable (P2)
**INTRODUCTION**			
Rationale	3	Describe the rationale for the review in the context of what is already known.	Introduction (P3)
Objectives	4	Provide an explicit statement of questions being addressed with reference to participants, interventions, comparisons, outcomes, and study design (PICOS).	Introduction (P4–5)
**METHODS**			
Protocol and registration	5	Indicate if a review protocol exists, if and where it can be accessed (e.g., Web address), and, if available, provide registration information including registration number.	Unpublished document circulated to collaborators (P5)
Eligibility criteria	6	Specify study characteristics (e.g., PICOS, length of follow-up) and report characteristics (e.g., years considered, language, publication status) used as criteria for eligibility, giving rationale.	Methods (P6)
Information sources	7	Describe all information sources (e.g., databases with dates of coverage, contact with study authors to identify additional studies) in the search and date last searched.	Methods (P5)
Search	8	Present full electronic search strategy for at least one database, including any limits used, such that it could be repeated.	Methods (P5)
Study selection	9	State the process for selecting studies (i.e., screening, eligibility, included in systematic review, and, if applicable, included in the meta-analysis).	Methods (P5)
Data collection process	10	Describe method of data extraction from reports (e.g., piloted forms, independently, in duplicate) and any processes for obtaining and confirming data from investigators.	Methods (P6)
Data items	11	List and define all variables for which data were sought (e.g., PICOS, funding sources) and any assumptions and simplifications made.	Methods (P6)
Risk of bias in individual studies	12	Describe methods used for assessing risk of bias of individual studies (including specification of whether this was done at the study or outcome level), and how this information is to be used in any data synthesis.	Methods (P7)
Summary measures	13	State the principal summary measures (e.g., risk ratio, difference in means).	Methods (P7)
Synthesis of results	14	Describe the methods of handling data and combining results of studies, if done, including measures of consistency (e.g., I^2) for each meta-analysis.	Methods (P7–8)
Risk of bias across studies	15	Specify any assessment of risk of bias that may affect the cumulative evidence (e.g., publication bias, selective reporting within studies).	Results (P7)
Additional analyses	16	Describe methods of additional analyses (e.g., sensitivity or subgroup analyses, meta-regression), if done, indicating which were pre-specified.	Methods<br>Results (P7)

RESULTS			
Study selection	17	Give numbers of studies screened, assessed for eligibility, and included in the review, with reasons for exclusions at each stage, ideally with a flow diagram.	Methods<br>Figure 1 (P8)
Study characteristics	18	For each study, present characteristics for which data were extracted (e.g., study size, PICOS, follow-up period) and provide the citations.	Table 1 (P8–9)
Risk of bias within studies	19	Present data on risk of bias of each study and, if available, any outcome level assessment (see item 12).	Figures 2–3 (P9)
Results of individual studies	20	For all outcomes considered (benefits or harms), present, for each study: (a) simple summary data for each intervention group (b) effect estimates and confidence intervals, ideally with a forest plot.	Figures 4–5 (P9)
Synthesis of results	21	Present results of each meta-analysis done, including confidence intervals and measures of consistency.	Table 2 (P9)
Risk of bias across studies	22	Present results of any assessment of risk of bias across studies (see Item 15).	Figure 9 (P9–10)
Additional analysis	23	Give results of additional analyses, if done (e.g., sensitivity or subgroup analyses, meta-regression [see Item 16]).	Tables 7–8, 9–10 (P10–11)
**DISCUSSION**			
Summary of evidence	24	Summarize the main findings including the strength of evidence for each main outcome; consider their relevance to key groups (e.g., healthcare providers, users, and policy makers).	Discussion (P12–15)
Limitations	25	Discuss limitations at study and outcome level (e.g., risk of bias), and at review-level (e.g., incomplete retrieval of identified research, reporting bias).	Discussion (P15)
Conclusions	26	Provide a general interpretation of the results in the context of other evidence, and implications for future research.	Discussion (P16)
**FUNDING**			
Funding	27	Describe sources of funding for the systematic review and other support (e.g., supply of data); role of funders for the systematic review.	Declared on online submission system (P17)

Supplementary Material 2. Search strategy for each database.

PubMed

(((((liver cancer [MeSH Terms])) OR (hepatoma[MeSH Terms])) OR (hepatic carcinoma [MeSH Terms])) OR (hepatocellular carcinoma [MeSH Terms])) OR (HCC)) AND ((((((((α-AFP [Title])) OR (fucosylated fraction of alpha-fetoprotein [Title])) OR (fucosylated fraction of α-fetoprotein [Title])) OR (alpha-fetoprotein [Title])) OR (α-fetoprotein [Title])) OR (AFP-L3 [Title])) OR (AFP)) OR (alpha-AFP))))).

Web of science

Step 1: liver cancer (Topic) or hepatoma (Topic) or hepatic carcinoma (Topic) or hepatocellular carcinoma (Topic) or HCC (Topic) Databases = WOS, BCI, KJD, MEDLINE, RSCI, SCIELO Timespan = All years.

Search language = Auto

Step 2: α-AFP (Topic) or fucosylated fraction of alpha-fetoprotein (Topic) or fucosylated fraction of α-fetoprotein (Topic) or alpha-fetoprotein (Topic) or α-fetoprotein (Topic) or AFP-L3 (Topic) or alpha-AFP (Topic).

Databases = WOS, BCI, KJD, MEDLINE, RSCI, SCIELO Timespan = All years

Search language = Auto

Step 3: #1 AND #2.

Databases = WOS, BCI, KJD, MEDLINE, RSCI, SCIELO Timespan = All years

Search language = Auto

Embase

Step 1: ' liver cancer ':ti,ab,kw OR ' hepatoma ':ti,ab,kw OR ' hepatic carcinoma ':ti,ab,kw OR ' hepatocellular carcinoma ':ti,ab,kw OR HCC:ti,ab,kw.

Step 2: α-AFP:ti,ab,kw OR ' fucosylated fraction of alpha-fetoprotein ':ti,ab,kw OR fucosylated fraction of α-fetoprotein:ti,ab,kw OR ' alpha-fetoprotein ':ti,ab,kw OR ' α-fetoprotein ':ti,ab,kw OR ' AFP-L3':ti,ab,kw OR ' alpha-AFP ':ti,ab,kw.

Step 3: #1 AND #2.

CNKI and Wanfang

(肝癌 OR HCC OR PHC) AND (AFP-L3 OR alpha-AFP OR α-AFP OR AFP OR 甲胎蛋白 OR 甲胎蛋白异质体).

Supplementary Material 3. The references list of study included in the meta-analysis.

1. Zhou B, Lu HB, Hou KZ. The relationship between serum Alpha-fetoprotein and alpha-fetoprotein isoplasts and the short-term efficacy and prognosis of hepatocellular carcinoma patients and their diagnostic value analysis. Int J Dig Dis. 2020; 40:63–66+70.
2. Wang XL, Huang QY, Wu YY. Clinical Diagnostic Value of Combined Detection of Alpha-fetoprotein Heterogenous Ratio and Serum Abnormal Prothrombin in Hepatocellular Carcinoma. Chin Foreign Med Res. 2020; 18:75–7.
3. Zhu YF, Xia JJ, Luo JJ, Zhu YF. Application of combined detection of alpha-fetoprotein alpha-fetoprotein heterogeneity and TNF-a factor in the diagnosis of primary liver cancer and its clinical value. J Clin Exp Med. 2020; 19:383–6.
4. Zeng FL, Wang DH, Su R, Yang HQ. Combined detection of AFP, AFP-L3 and DKK1 in the diagnosis of primary liver cancer. J North Sichuan Med College. 2020; 35:130–2.
5. Jiao SJ. The diagnostic value of serum DCP, GP73, AFP-L3 in primary liver cancer. Henan Med Res. 2019; 28:3980–1.
6. Wang XM. The value of serum DCP, GP73, AFP-L3 combined detection in patients with primary liver cancer. Inter Mongolia Med J. 2019; 51:720–2.
7. Zhang Q, Deng RF. The clinical significance of alpha-fetoprotein isoplastids in the diagnosis of hepatocellular carcinoma. Chin Comm Doc. 2019; 35:125.
8. Han SG, Huang CY, Zheng X, Liu HM, Zhou XY, Li SL. Comparison of the diagnosis value of enzyme linked immunosorbent assay method and enzyme linked immunosorbent assay on detected Alpha fetal protein heterogeneity for primary liver cancer. Chin J Clin Pharmacol. 2018; 34:1517–9.
9. Li M. The study of diagnostic value of alpha fetoprotein heteroplasm detection in patients with liver cancer and liver disease. J Baodou Med College. 2017; 33:54–5.
10. Cheng T, Liu XH, Zhang QP, Tang ZL, Song ZL. The value of detecting a-Fetoprotein variants with a micro-spin column method in differetnial diagnosis of hepatocellular carinoma and benign liver diseases. Chin J Lab Diagn. 2017; 21:948–50.
11. Zhou YJ. Application value of alpha-fetoprotein isoplast in early diagnosis of hepatocellular carcinoma. Shenzhen Inte Chin West Med. 2015; 25:78–9.
12. Lu AH, Song YH, Dong XP. The diagnostic value of alpha-fetoprotein isoplasts in hepatocellular carcinoma. Chin J Integr Trad West Med. 2014; 23:423–4.
13. Li HY, Bao SJ. Significance of Alpha-feto-protein Heterogeneity Detection in Diagnosis for Liver Cancer. Chin Oncol. 2013; 22:406–8.
14. Cao XZ. Observation on the efficacy of alphafetoprotein isoplast detection in hepatocellular carcinoma with peritoneal effusion. Guide Chin Med. 2013; 11:283–4.
15. Chen Y, LinYY, Hu MH, Chen YS. Significance of Lens culinaris reactive alpha-fetoproteins in early diagnosis and warning of hepatocellular carcinoma. Modern Oncol. 2012; 20:1652–4.
16. Han SG, Li GA, Huang CY, Zhao ZH, Zhou XY, Li SL. Value of alpha fetoprotein isoforms in diagnosis of hepatocellular carcinoma with ACSC. Chin J Health Lab Tech. 2012; 22:1348–9.
17. Huang CY, Han SG, Zhang JH, Wang XR, Li SL, Zhou XY. Clinical research of AFP variant tested with enzyme linked immunosorbent assay and microcentrifugal column method. Modern Oncol. 2012; 20:2574–6.
18. Ma DY, Yu L, Feng JK, Sheng L. Differential diagnosis of hepatic carcinoma and detect Alpha-fetopreteins heterogeneity (AFP-L3) using microspincolumn coupled with Lens culinaris agglutinin. Conte Med. 2011; 17:113–4.
19. Ji J, Gu X, Gao Z, Sun XJ, Dong GL, Zhou J. Application evaluation of apha-fetoprotein variant with lectin microcentrifugal column method in the diagnosis of primary liver carcinoma. Lab Med. 2011; 26:256–9.
20. Toyoda H, Kumada T, Tada T, Kaneoka Y, Maeda A, Kanke F, Satomura S. Clinical utility of highly sensitive Lens culinaris agglutinin-reactive alpha-fetoprotein in hepatocellular carcinoma patients with alpha-fetoprotein <20 ng/mL. Cancer Sci. 2011; 102:1025–31. https://doi.org/10.1111/j.1349-7006.2011.01875.x PMID:21244578
21. Nouso K, Kobayashi Y, Nakamura S, Kobayashi S, Takayama H, Toshimori J, Kuwaki K, Hagihara H, Onishi H, Miyake Y, Ikeda F, Shiraha H, Takaki A, et al. Prognostic importance of fucosylated alpha-fetoprotein in hepatocellular carcinoma patients with low alpha-fetoprotein. J Gastroenterol Hepatol. 2011; 26:1195–200. https://doi.org/10.1111/j.1440-1746.2011.06720.x PMID:21410750

22. Jia ZL, Wang L, Liu C, Zhang HY, Zhai LN, Yu ZH. The clinical significance of Alpha-fetoprotein L3 in diagnosis of hepatocellular carcinoma. Chin Oncol. 2010; 19:686–8.

23. Niu XM, Liang P, Fu HR. Clinical valur of detection apha-fetoprotein heterogentiy for differential diagnosis in begigan and maligant liver disease. COccup Health. 2010; 26:707–9.

24. Tamura Y, Igarashi M, Kawai H, Suda T, Satomura S, Aoyagi Y. Clinical advantage of highly sensitive on-chip immunoassay for fucosylated fraction of alpha-fetoprotein in patients with hepatocellular carcinoma. Dig Dis Sci. 2010; 55:3576–83. https://doi.org/10.1007/s10620-010-1222-5 PMID:20407827

25. Zheng Y, Shan XJ, Ren MR, Ding WJ, Pei LH. The significance of detection of alpha-fetoprotein isoplasts in the diagnosis of primary hepatocellular carcinoma. Zhejiang Prac Med. 2009; 14:3–4.

26. Y J, Xie Y, Gong H, Mu CL. Correlation of concentration of AFP-L3 markers with hepatocellular carcinoma. Chin Tropical Med. 2009; 9:292–3.

27. Sun GZ, Zhao XY, Li JH, Zhao GQ, Wang SX, Kong SL. [Detection of alpha-fetoprotein-L3 using agglutinin-coupled spin column to be used in diagnosis of hepatocellular carcinoma]. Zhonghua Yi Xue Za Zhi. 2008; 88:1986–8. PMID:19062741

28. Wang YZ, Wu GX, Chen M, Pu XK, Hang SR, Ruan L. Application of serum AFP-L3 variants assay f or differential diagnosis of liver disease. Jiangsu Med. 2007; 33:565–7.

29. Xu AF, Wang MC, Sui DM, Yuan YH, Chen G, Lou GQ. [Subject diagnostic value of detecting a1pha-fetoprotein variants with a new microspincolumn method in hepatocellular carcinoma]. Zhonghua Shi Yan He Lin Chuang Bing Du Xue Za Zhi. 2007; 21:67–9. PMID:17429541

30. Sterling RK, Jeffers L, Gordon F, Venook AP, Reddy KR, Satomura S, Kanke F, Schwartz ME, Sherman M. Utility of Lens culinaris agglutinin-reactive fraction of alpha-fetoprotein and des-gamma-carboxy prothrombin, alone or in combination, as biomarkers for hepatocellular carcinoma. Clin Gastroenterol Hepatol. 2009; 7:104–13. https://doi.org/10.1016/j.cgh.2008.08.041 PMID:18849011

31. Durazo FA, Blatt LM, Corey WG, Lin JH, Han S, Saab S, Busuttil RW, Tong MJ. Des-gamma-carboxyprothrombin, alpha-fetoprotein and AFP-L3 in patients with chronic hepatitis, cirrhosis and hepatocellular carcinoma. J Gastroenterol Hepatol. 2008; 23:1541–8. https://doi.org/10.1111/j.1440-1746.2008.05395.x PMID:18422961

32. Zinkin NT, Grall F, Bhaskar K, Otu HH, Spentzos D, Kalmowitz B, Wells M, Guerrero M, Asara JM, Libermann TA, Afdhal NH. Serum proteomics and biomarkers in hepatocellular carcinoma and chronic liver disease. Clin Cancer Res. 2008; 14:470–7. https://doi.org/10.1158/1078-0432.CCR-07-0586 PMID:18223221

33. Leerapun A, Suravarapu SV, Bida JP, Clark RJ, Sanders EL, Mettler TA, Stadheim LM, Aderca I, Moser CD, Nagorney DM, LaRusso NF, de Groen PC, Menon KV, et al. The utility of Lens culinaris agglutinin-reactive alpha-fetoprotein in the diagnosis of hepatocellular carcinoma: evaluation in a United States referral population. Clin Gastroenterol Hepatol. 2007; 5:394–402. https://doi.org/10.1016/j.cgh.2006.12.005 PMID:17368240

34. Shimizu A, Shiraki K, Ito T, Sugimoto K, Sakai T, Ohmori S, Murata K, Takase K, Tameda Y, Nakano T. Sequential fluctuation pattern of serum des-gamma-carboxy prothrombin levels detected by high-sensitive electrochemiluminescence system as an early predictive marker for hepatocellular carcinoma in patients with cirrhosis. Int J Mol Med. 2002; 9:245–50. PMID:11836630
